# Effect of media and fermentation conditions on surfactin and iturin homologues produced by *Bacillus natto* NT-6: LC–MS analysis

**DOI:** 10.1186/s13568-019-0845-y

**Published:** 2019-07-27

**Authors:** Dongfang Sun, Jianmeng Liao, Lijun Sun, Yaling Wang, Ying Liu, Qi Deng, Ning Zhang, Defeng Xu, Zhijia Fang, Wenjing Wang, Ravi Gooneratne

**Affiliations:** 1 College of Food Science and Technology, Guangdong Ocean University, Guangdong Provincial Key Laboratory of Aquatic Product Processing and Safety, Key Laboratory of Advanced Processing of Aquatic Products of Guangdong Higher Education Institution, NO. 1 Haida Road, Mazhang District, Zhanjiang, 524088 Guangdong Province China; 2 National Marine Products Quality Supervision & Inspection Center, Mazhang District, Zhanjiang, 524000 China; 3 Department of Wine, Food and Molecular Biosciences, Faculty of Agriculture & Life Sciences, Lincoln University, Lincoln, 7647 Canterbury New Zealand

**Keywords:** LC–MS, Antimicrobial lipopeptide, *Bacillus natto*, Surfactin, Iturin

## Abstract

Lipopeptides possess excellent broad spectrum antimicrobial activity. Different lipopeptides have their own unique chemical structures, properties and biological activities. Quantitative analysis of the lipopeptides iturin and surfactin and their homologues produced by *Bacillus natto* NT–6 subjected to different culture media, shaking speed of rotary shaker, and liquid and solid fermentation methods was conducted using LC–MS. For iturins, liquid-state fermentation in Landy medium at a shaking speed of 160 r min^−1^ was the most suitable for maximal homologue production. Addition of 0.4% attapulgite powder increased production by 1.92-fold; activated carbon significantly reduced production. For surfactin homologues, solid-state fermentation in potato dextrose broth medium at shaking speed > 160 r min^−1^ was the best. Addition of 0.4% attapulgite powder increased production by 1.96-fold; activated carbon had no effect. Thus it is clear that fermentation conditions can be manipulated to maximize iturin and surfactin production.

## Introduction

In *Bacillus subtilis* spore metabolites, the lipopeptides consist of a lipophilic fatty acid chain and a hydrophilic peptide ring and the most common are surfactin, iturin, and fengycin (Banat et al. 2010). These three lipopeptides have gained wide attention because they possess antibacterial and biosurfactant properties and therefore have the potential to be useful in the fields of medicine, agriculture, food, cosmetics, petroleum, mining and environmental governance (Desai and Banat 1997; Banat et al. 2010; Marchant and Banat 2012).

Surfactin, iturin, and fengycin have several homologues (Stein 2005; Shaligram and Singhal 2010) with unique structure, chemical properties and biological activities (Stein 2005; Banat et al. 2010). For example, surfactin produces a strong inhibitory effect on bacteria (Chen et al. 2015), iturin has antifungal activity (Sandrin et al. 1990) and fengycin inhibits growth of mold (Bie et al. 2006). The production of these lipopeptides is markedly affected by components in the growth medium (Sun et al. 2013). The antimicrobial lipopeptides are synthesized by a non-ribosomal peptide synthetase by a method called a “multi-carrier thiotemplate mechanism” (Schneider and Marahiel 1998; Koumoutsi et al. 2004). The amino acid sequence of the products is determined by the sequence of the peptide synthetase modules (Stein 2005) and each module contains multiple functional domains. The production of multiple homologues of lipopeptides by a complex multicomponent enzyme system is affected by the experimental conditions (Shaligram and Singhal 2010). Therefore, in order to maximize lipopeptide yield, it is important to analyze the effects of different fermentation conditions on the yield and composition of antimicrobial lipopeptides. The effect of the growth media and fermentation conditions for optimal antimicrobial lipopeptide synthesis has been previously investigated (Ohno et al. 1995; Thompson et al. 2001; Nitschke et al. 2004). Ohno et al. (1995) demonstrated that the ratio between iturin and surfactin is affected by temperature. Koumoutsi et al. (2004) showed that the stage of microorganism growth affects the quantity of antimicrobial lipopeptides. However, previous studies have all used HPLC for qualitative analysis, which cannot precisely quantify the lipopeptides homologues.

Previous studies have shown that fermentation conditions are critical to the production of lipopeptide (Shaligram and Singhal 2010; Mnif et al. 2012). However, due to the restriction of the synchronous quantitative testing method of the lipopeptide component, there is still no detailed analysis of the composition of the mixture of lipopeptides and effects on the content of their homologues. This study, based on the constructed LC–MS qualitative analysis method (Deng et al. 2017), identified optimized experimental conditions (different media, temperature, ventilation and fermentation methods) to produce maximal lipopeptides concentrations from *Bacillus natto NT*-*6* using LC–MS, expecting to achieve the directional accumulation of specific components in antimicrobial lipopeptides and lay the foundation of the production of specific efficacy lipopeptide.

## Materials and methods

### Microorganism and culture medium

The wild-type organism *Bacillus subtilis* subsp. *natto* NT-6 obtained from China General Microbiology Culture Collection Center (CGMCC 8121) was used in this study.

### Preparation of antibacterial lipopeptides

For preparation of the seed culture, *B. natto* NT-6 colonies from the liquid broth were grown in 250-mL flasks containing 80 mL of BPY liquid medium containing beef extract, 5.0 g; peptone, 10.0 g; yeast extract, 5.0 g; glucose, 10.0 g; NaCl, 5.0 g; and distilled water, 1000 mL; with pH set to 7.2.

Liquid fermentation for antibacterial lipopeptides and its extraction: To produce lipopeptides, 5% (v/v) of seed culture was inoculated into a 250-mL shake flask containing 80 mL of sterile Landy medium and cultivated at 28 °C with shaking at 160 r min^−1^ for 36 h. Next, the culture was centrifuged at 10,000 r min^−1^ for 15 min to remove bacterial cells and impurities, and the supernatant was collected. The supernatant pH was adjusted to 2 with formic acid and then left overnight at 28 °C. The pellet formed after centrifugation at 10,000 r min^−1^ for 20 min was dissolved in methanol and the resultant solution was adjusted to pH 7.0 and centrifuged again at 10,000 r min^−1^ for 20 min. The supernatant was concentrated using a rotary evaporator at 60 °C and extracted three times with methanol. The resultant solution containing the lipopeptides collected was stored at 4 °C.

The solid-state fermentation medium was inoculated with 10% (v/w) seed culture and mixed under sterile conditions. The solid-state fermentation was carried out at 28 °C for 72 h. The fermentation product was dried at 50 °C and then extracted with methanol (w/v = 1:5) for 3 h and filtered through gauze. Next, the solution was centrifuged at 10,000 r min^−1^ for 15 min to remove bacterial cells and impurities, and the supernatant collected was stored at 4 °C.

### Experimental design

Landy medium (glucose, 10.0 g; L-monosodium glutamate, 5.0 g; MgSO_4_, 0.5 g; KCl, 0.78 g; KH_2_PO_4_, 1.0 g; FeSO_4_, 0.05 mg; MnSO_4_, 5.0 mg; CuSO_4_, 0.16 mg; and distilled water, 1000 mL; pH 7.2), potato dextrose broth (PDB) medium (potato, 200 g; glucose, 20 g; and distilled water, 1000 mL), nutrient broth (NB) medium (peptone, 10.0 g; beef extract powder, 3.0 g; NaCl, 5.0 g and distilled water, 1000 mL; pH 7.2) and LB medium (Tryptone, 10.0 g; yeast extract, 5.0 g; NaCl, 10.0 g; pH 7.0) were used as culture media to produce antimicrobial lipopeptides.

The effects of shaking speed of rotary shaker (speeds were designated as 120 r min^−1^, 140 r min^−1^, 160 r min^−1^, 180 r min^−1^, 200 r min^−1^, respectively), solid-state fermentation (wheat bran 70%, bean pulp 28%, 1.20% glucose, 0.55% L-sodium glutamate, 0.15% (NH_4_) _2_SO_4_, 0.10% KH_2_PO_4_·3H_2_O, ratio of material to water of 5:3, fermented for 48 h in 10 g/250 mL flasks under 28 °C) and liquid fermentation (Landy medium, 28 °C, shaking speed of 160 r min^−1^, 36 h) on the production and composition of antibacterial lipopeptides were tested (three parallel tests). Either 0.6% activated carbon powder or 0.4% attapulgite powder was added to the Landy medium and fermentate for 36 h at 28 °C, at a shaking speed of 160 r min^−1^. Next the samples obtained from different fermentation conditions were extracted, processed, and the concentration of iturin and surfactin and its homologues were determined by LC–MS.

### LC–MS conditions

Surfactin and iturin analyses were performed on a Thermo Scientific Surveyor HPLC system composed of a Surveyor MS pump Plus, with an on-line degasser and a Surveyor auto sampler Plus coupled to a Thermo TSQ Quantum Access tandem mass spectrometer equipped with an electrospray ionization (ESI) source (LC–MS, Thermo, USA) as described by (Deng et al. 2017). An aliquot of 10 μl of sample was injected into a reversed-phase chromatographic column: Venusil XBP CN (150 × 2.1 mm, of 100 Å pore diameter and particle size 5 μm; Thermo, USA). The column was thermo-stabilized at 35 °C for separation with a flow rate of 8.0 μl min^−1^. A mobile phase of 5 mmol L^−1^ ammonium acetate solution containing 0.1% (v/v) formic acid (A) and acetonitrile (B) was used. The following linear gradient elution was used: 60% A at 0 min, increased to 10% A from 0 to 4 min, held at 10% A from 4 to 7 min, then decreased to 60% A from 7 to 7.1 min, and further held at 60% A until 12 min. The flow-rate was set at 250 μl min^−1^.

The mass spectrometer was operated in the positive ion mode using ESI under the following conditions: spray voltage: 4500 V, sheath gas pressure: 40 au, auxiliary gas pressure: 15 au, blowback pressure: 0.5 psi, ion source temperature: 350 °C, capillary temperature: 270 °C, and collision pressure: − 22 mTorr. Acquisition was performed in the selected ion monitoring (SIM) mode.

### Statistical analysis

SPASS Statistics 21 was used to analyze the data and the Microsoft Excel program to prepare the graphics. The results are expressed as mean ± S.D.

## Results

### Effects of different fermentation medium for antimicrobial lipopeptides production and composition

**Fig. 1:**
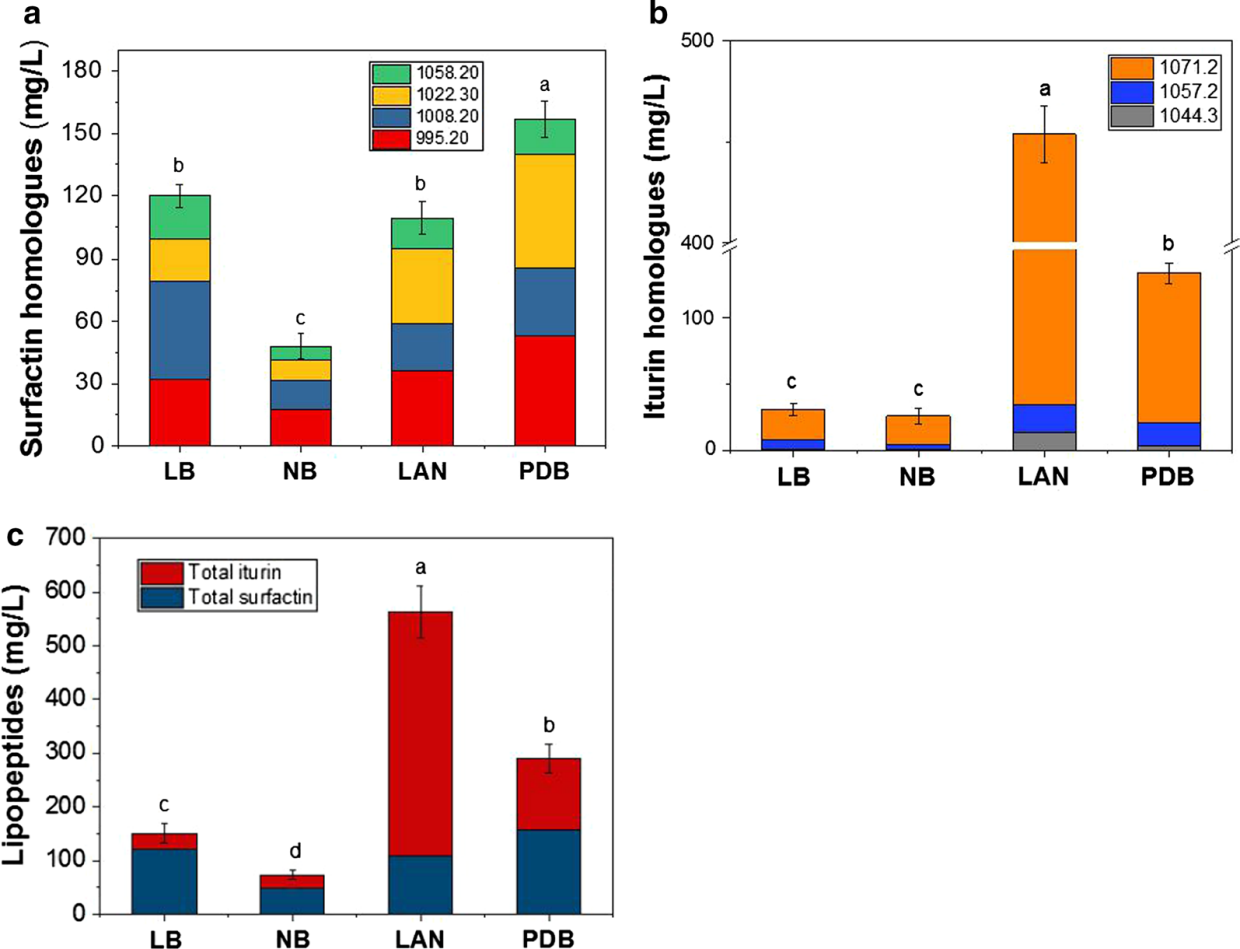
Effect of different culture media on the production of surfactin homologues (**a**), iturin homologues (**b**) and lipopeptides (**c**). Values are expressed as mean ± SD, n = 4. The different letters indicate significant differences between treatments (p < 0.05). *LB* lysogeny broth, *NB* nutrient broth, *LAN* Landy medium, *PDB* potato-dextrose broth

As shown in Fig. 1b, the iturin produced in fermentation media was composed of three homologues with mass–charge ratios (m/z) of 1044.30, 1057.20 and 1071.20. Among them, the m/z 1071.20 component accounted for > 80% and was highest with the Landy medium. The surfactin homologues had m/z of 995.20, 1008.20, 1022.30, and 1058.20 and with Landy and PDB media, the main components had m/z of 995.20 and 1022.30. However with the LB and NB media, the largest proportion of homologues had m/z of 1008.20 and 995.20, respectively. Thus, unlike with iturin, the type of culture medium largely influenced the surfactin homologue proportion.

Fermentation medium is one of the most important factors that influence lipopeptide production. As shown in Fig. 1, the production levels of total lipopeptides and its homologues from the common media (Landy medium, PDM, NB, LB) were significantly different (p < 0.05). Among the media tested, Landy medium was the most effective with a total lipopeptide production of 563.20 mg L^−1^, significantly higher than with other cultures (p < 0.01) including the next best medium, the PDM (289.82 mg L^−1^).

### Effect of shaking speed on antimicrobial lipopeptide production and composition

**Fig. 2:**
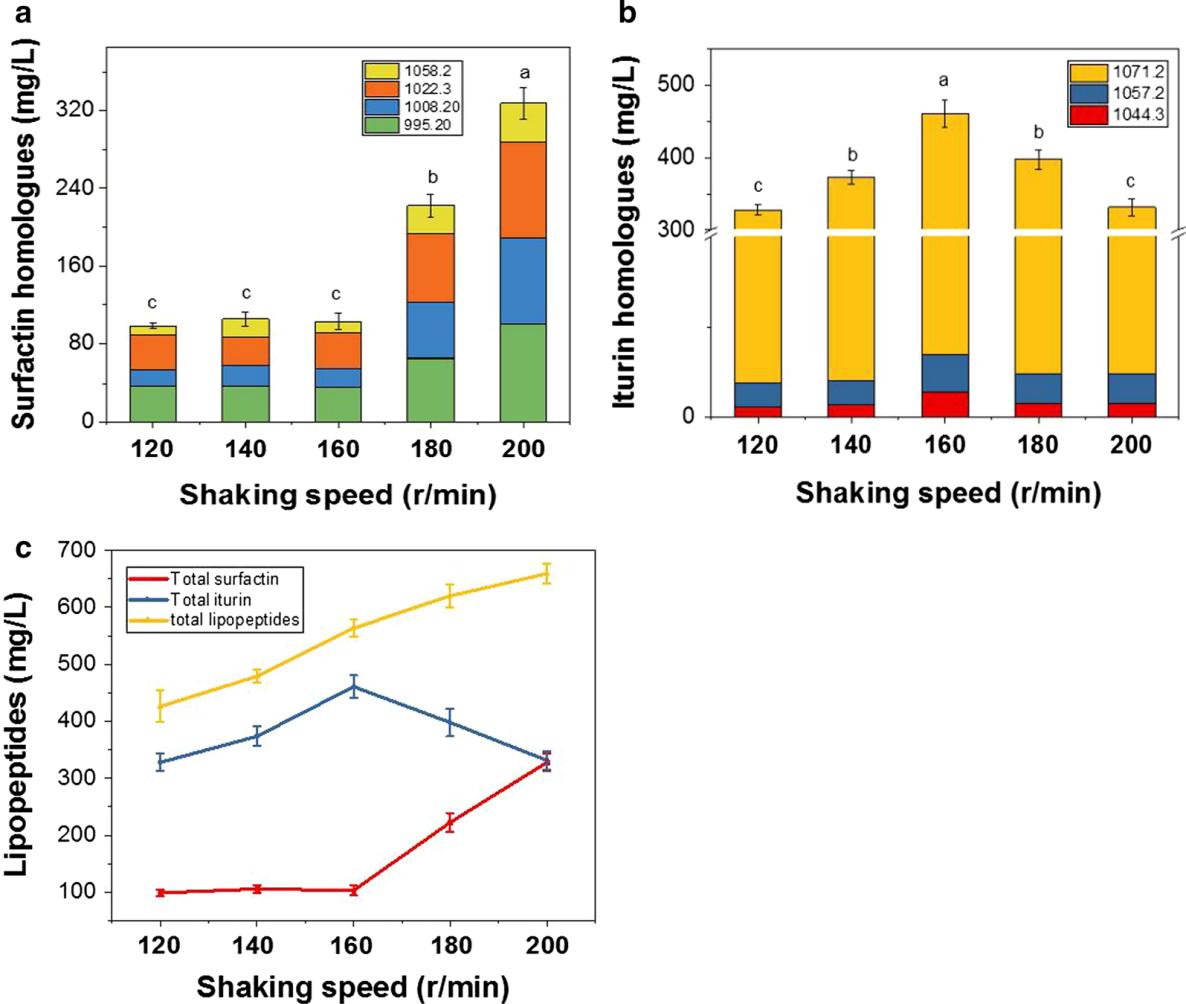
Concentration of surfactin homologues (**a**), iturin homologues (**b**) and lipopeptide (**c**) production at different shaking speeds. Values are expressed as mean ± SD, n = 5. The different letters indicate significant differences between treatments (p < 0.05)

The shaking speed of the rotary shaker was adjusted to between 120 and 200 r min^−1^. The increase in shaking speed increased the lipopeptide concentration with maximal concentration (659.00 mg L^−1^) at 200 r min^−1^ (Fig. 2).

The content and percentage of each component in antimicrobial lipopeptides also varied with shaking frequency (Fig. 2). The proportion of iturin: surfactin was highest (81.78%) at 160 r min^−1^ but declined at higher speeds because of an increase in the surfactin concentration.

### Effect of different fermentation types on lipopeptides components

In Landy medium when *B. natto* was grown at 28 °C and 160 r min^−1^ for 36 h, the lipopeptide production was 657.23 mg L^−1^. Of this, iturin components accounted for 289.18 mg L^−1^ (~ 44% of the total), and was constituted mostly of homologues with m/z of 1044.30, 1057.20 and 1071.20 (which accounted for ~ 26.5%, 48.6% and 24.8%, respectively). Surfactin components production (368.05 mg L^−1^) accounted for 56.00% of the total, and was constituted mostly of homologues with m/z 995.20, 1008.20, 1022.30 and 1058.20 (which accounted for ~ 1.6%, 14.0%, 53.7% and 30.7%, respectively) (Table 1).

**Table 1:** Effect of different culture media on lipopeptide production

	[M+H]^+^	LF	Percentage	SSF	Percentage
Surfactin isoforms	995.20	5.89 ± 0.16	1.60 ± 0.04	39.06 ± 0.68	1.24 ± 0.02
	1008.20	51.63 ± 1.61	14.03 ± 0.44	501.17 ± 7.50	15.91 ± 0.24
	1022.30	197.54 ± 3.12	53.68 ± 0.85	1334.85 ± 15.35	36.02 ± 0.41
	1058.20	122.93 ± 2.43	30.69 ± 0.61	1322.31 ± 15.11	41.97 ± 0.48
Iturin isoforms	1044.30	76.72 ± 1.88	26.53 ± 0.65	105.95 ± 0.51	8.63 ± 0.04
	1057.20	140.67 ± 3.54	48.66 ± 1.25	631.39 ± 8.32	51.43 ± 0.68
	1071.20	71.75 ± 1.67	24.81 ± 0.58	490.33 ± 5.49	39.94 ± 0.45
Total surfactin		368.05 ± 6.33	56.00 ± 0.96	3150.61 ± 34.66	71.96 ± 0.79
Total iturin		289.18 ± 4.26	44.00 ± 0.65	1227.67 ± 13.85	28.04 ± 0.31
Total lipopeptide		657.23		4378.28	

*LF* liquid fermentation, *SSF* solid state fermentation

In the solid-state fermentation medium, when *B.* *natto* was cultured at 28 °C for 48 h and the ratio of medium material to water was 5:3, the lipopeptide production was 4.3782 g kg^−1^. Of this, iturin components accounted for 28.0% of the total, and was constituted mostly of homologues with m/z of 1044.30, 1057.20 and 1071.20 (which accounted for ~ 8.6%, 51.4% and 39.9%, respectively). In contrast, the surfactin components accounted for ~ 72.0% of the total, and were constituted mostly of homologues with m/z of 995.20, 1008.20, 1022.30 and 1058.20 (which accounted for ~ 1.2%, 15.9%, 36.0% and 42%, respectively).

### Effect of solid matrix with large specific surface area addition on antimicrobial lipopeptide components

**Fig. 3:**
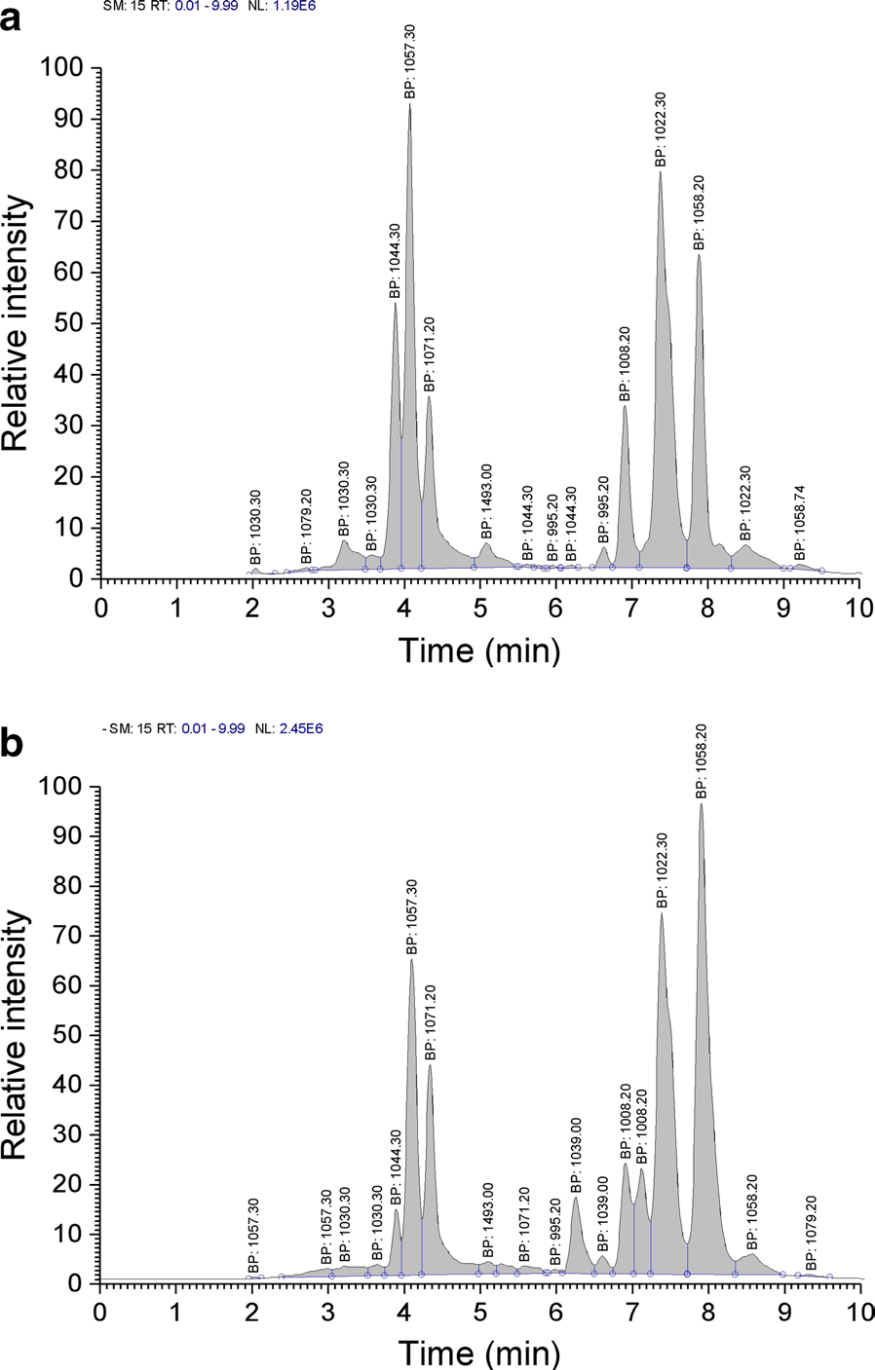
Ion chromatogram of iturin and surfactin homologues produced by liquid-state fermentation (**a**), and solid-state fermentation (**b**)

When 0.6% activated carbon powder in the Landy medium and *B.* *natto* was grown at 28 °C, 160 r min^−1^ for 36 h, the lipopeptide production (932.72 mg L^−1^) was 1.42-fold that without activated carbon (Fig. 3). The iturin components production (145.04 mg L^−1^) was > 2-fold than without activated carbon, accounting for ~ 15.6% of the total, and was constituted mostly of homologues with m*/z* 1044.30, 1057.20, and 1071.20 (which accounted for 23.6%, 41.0% and 35.4%, respectively) while it significantly reduced the proportion of m/z 1071.20 (p < 0.05). When activated carbon was added, the surfactin concentration (787.68 mg L^−1^) was 2.14-fold higher than without activated carbon, accounting for ~ 84.5% of the total lipopeptide production, and was constituted mostly of homologues with m/z of 995.20, 1008.20, 1022.30 and 1058.20 (which accounted for ~ 1.5%, 15.1%, 51.5% and 31.9% respectively). The proportion of each homologue component was similar both with and without activated carbon. Thus it was apparent that surfactin synthesis was promoted by the addition of activated carbon while synthesis of iturin was inhibited by it.

**Fig. 4:**
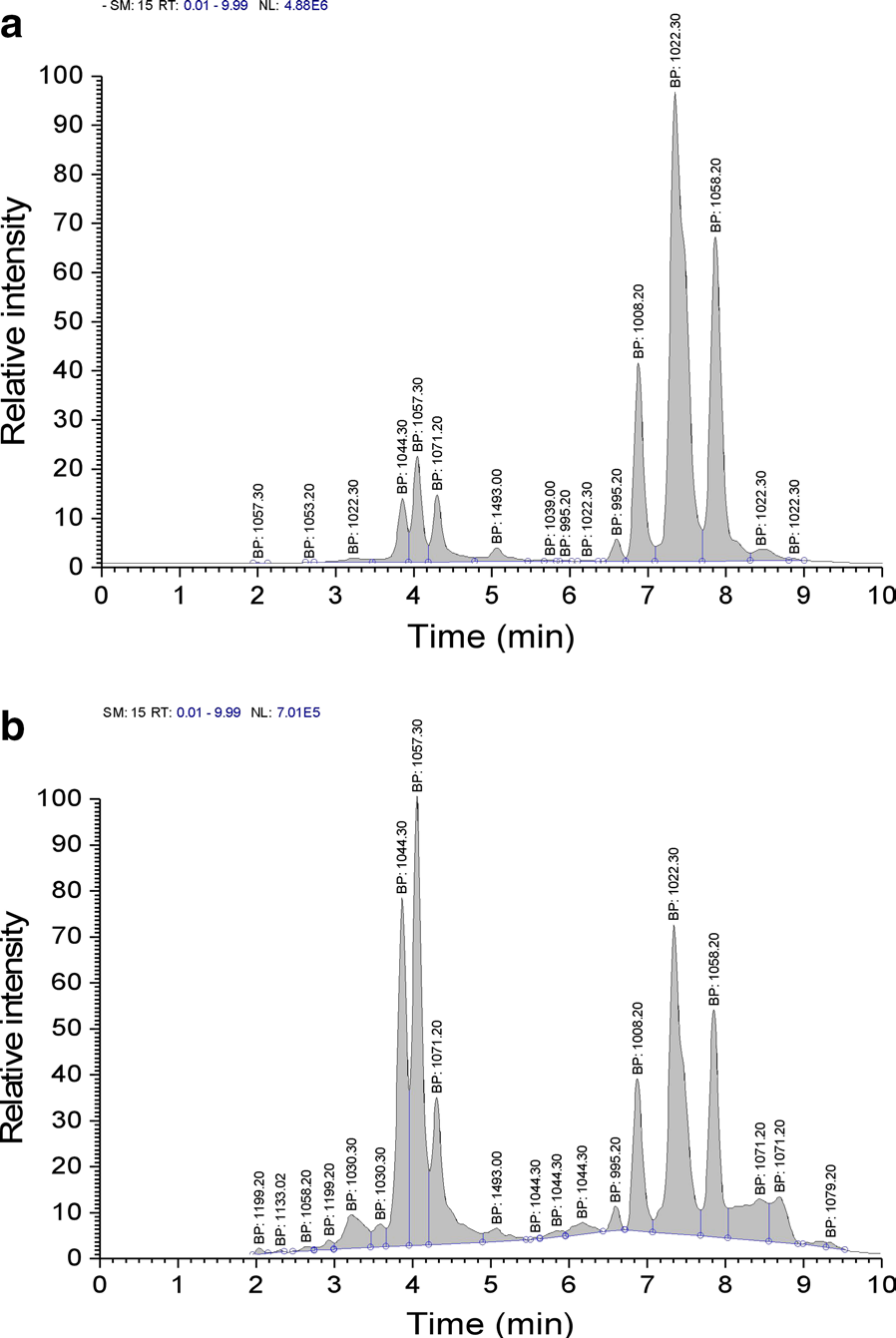
Ion chromatogram of iturin and surfactin homologues produced by Landy medium with added 0.6% activated carbon powder (**a**) and 0.4% attapulgite powder (**b**)

Adding 0.4% attapulgite powder to Landy medium at 28 °C, and with a shaking speed of 160 r min^−1^ for 36 h, the total lipopeptide production was ~ 1.94-fold (1276.7 mg L^−1^) higher than in the control group (without attapulgite). As shown in Fig. 4, the iturin production (554.58 mg L^−1^) was ~ 1.92-fold more than without attapulgite and accounted for ~ 43.4% of the total production, and was constituted mostly of homologues with m/z of 1044.30, 1057.20 and 1071.20 (which accounted for ~ 4.9%, 64.5% and 30.6%, respectively). Compared with the control group, the proportion of the 1044.30 homologue was reduced but the proportions of the 1057.20 and 1071.20 components were significantly increased (p < 0.05). The surfactin concentration (722.08 mg L^−1^) was ~ 1.96-fold more than in the control group, and accounted for ~ 56.6% of total lipopeptide production, constituted mostly of homologues with m/z of 995.20, 1008.20, 1022.30 and 1058.20 (which accounted for ~ 2.3%, 17.4%, 53.2% and 27.2%, respectively) with no significant change in the m/z charge ratios compared with the control group.

## Discussion

Previous studies have shown that fermentation conditions are critical to lipopeptide production (Shaligram and Singhal 2010; Mnif et al. 2012). However, because of the lack of accurate methods to quantify lipopeptide homologues, there is no detailed analysis of the effect of fermentation conditions on the composition of lipopeptide homologues. This study, based on a LC–MS method (Deng et al. 2017), identified optimized experimental conditions (different media, temperature, shaking speed, fermentation methods) to produce maximal lipopeptide concentrations from *Bacillus natto* NT-6.

It is clear that media is the most important factor that affects the production of lipopeptides and abundance of nutrients is not conducive to the lipopeptide synthesis which is agreement with a previous report (Akpa et al. 2001; Stein 2005; Andrade et al. 2016). Landy medium is generally regarded as a good medium for the production of lipopeptides, and has often been used in previous studies (Koumoutsi et al. 2004; Fahim et al. 2012) but maximal production also depends on temperature and the shaking speed. It was apparent from this study that with Landy medium, at 30 °C and shaking speed of 160 r min^−1^, iturin yield was highest (453.90 mg L^−1^), significantly higher than with other media tested (p < 0.05). The iturin component made up 80%, and surfactin 20%, which demonstrated that Landy medium is the most suitable culture medium for iturin production. A high percentage (92%) of iturin production was of the m/z 1071.20 type and hence possessed only a narrow antibacterial spectrum. Thus, a high lipopeptide yield does not always increase the spectrum of functionality. Although the PDB medium is often used for mold cultivation, our study showed that the yield of surfactins produced by the NT-6 strain in PDB medium (156.81 mg L^−1^) was significantly higher than that produced in the Landy medium (109.33 mg L^−1^) but in this medium the yield of iturins was low (p < 0.05). It demonstrated that PDB is better suited for the synthesis of surfactins and this has not been reported previously.

It has been shown that the high dissolved oxygen achieved at high shaking speed plays an important role in the synthesis of surfactin (Shaligram and Singhal 2010; Fahim et al. 2012). These studies shown that increasing shaking speed could promote lipopeptide production. In contrast, iturin production, which also increased continuously with shaking speed, was maximal at 160 r min^−1^ and accounted for 81.78% of the total production. But iturin production decreases as the speed increases when the speed is over 160 r min^−1^. This is in contrast to a previous report (Fahim et al. 2012).

Surfactin production did not change much between shaking speeds of the rotary shaker of 120–160 r min^−1^ but increased significantly at shaking speeds > 160 r min^−1^ (p < 0.05). The changes in shaking speed did not significantly affect the relative proportions of iturin and surfactin. During industrial production, a high dissolved oxygen concentration was achieved by increasing the air flux and the stirring rate in the fermentor. The dissolution and transfer of oxygen in fermentation broth was probably inhibited by the great deal of foam produced during high-speed air flux and stirring. Also, leakage of liquid from the fermentor can easily occur during high-speed air flux and stirring in the fermentation process of surfactant-type lipopeptide compounds with strong surface activity, which markedly reduces the fermentation efficiency of the fermentor (Fahim et al. 2012; Alonso and Martin 2016). Therefore, in industrial production, high-speed air flux and stirring may not achieve high yields of lipid peptide production. Therefore, it is necessary to explore novel methods to solve the limitation on surfactin and iturin production caused by increasing air flux and stirring.

**Fig. 5:**
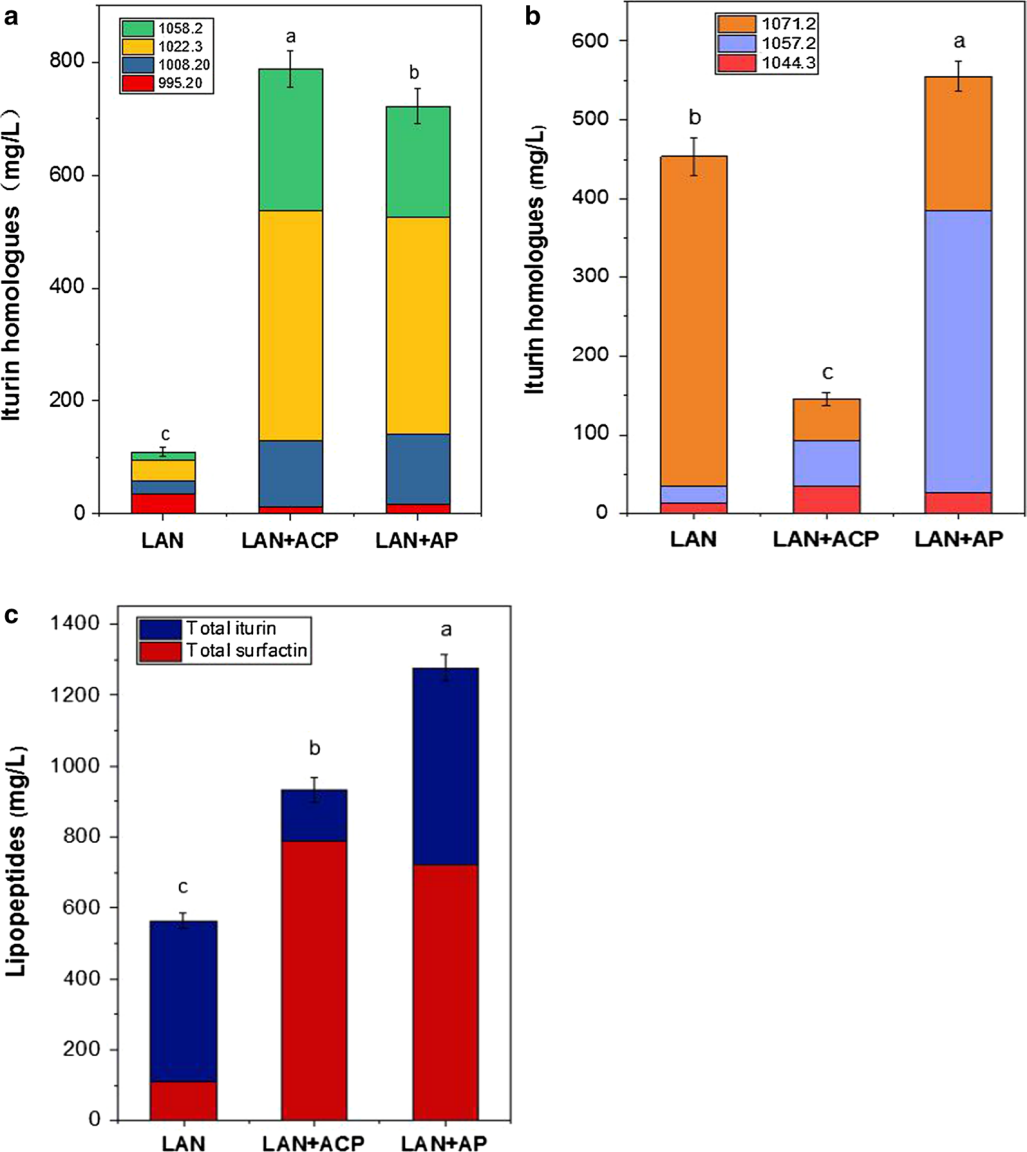
Effect of activated carbon powder (ACP) and attapulgite powder (AP) addition to Landy medium on surfactin homologue concentrations (**a**), iturin homologue production (**b**), and lipopeptides production (**c**). Values are mean ± SD, n = 3. The different letters indicate significant differences between treatments (p < 0.05)

Previous studies (Ohno et al. 1995; Nitschke et al. 2004; Zohora et al. 2013; Mnif et al. 2012) have mostly focused on the impact of solid or liquid fermentation on the production of iturin or surfactin, and the effect of fermentation conditions on the composition of different lipopeptides has not been reported. As reported above, we compared the composition of lipopeptides produced by the two fermentation methods (solid vs liquid) and observed significant differences in the type and yield of the lipopeptides and their homologue constituents (p < 0.05). The total production of solid-state fermented lipopeptides was ~ 66-fold greater than by liquid fermentation. Under liquid fermentation conditions, iturin and surfactin accounted for ~ 44% and 56%, respectively compared to 28% and 72% with the solid-state fermentation medium. Compared with the liquid fermentation method, a new homologue component with m/z 1039.00 was evident under solid-state-fermentation conditions, and two homologues of the homologous component with m/z 1008.20 were observed in the chromatograms (Fig. 5b). In addition, iturin homologue component with m/z 1044.30 made up a significantly lower proportion under solid-state fermentation conditions than with liquid fermentation (p < 0.05); the surfactin proportion of the 1022.30 component was reduced but the proportion of the 1058.20 component was significantly increased (p < 0.05). These results confirmed that different fermentation methods can markedly affect the composition and concentration of the lipopeptide components. Liquid fermentation was ideal for production of iturin components, while solid-state fermentation was more suited for surfactin. Thus different fermentation methods need to be chosen for maximal production of different lipopeptides.

Addition of a solid matrix in liquid-state fermentation could lead to biofilm formation. It has been shown that the yields of some metabolic products are increased when biofilms are formed on solid surfaces (Zohora et al. 2009). Shahedur and Takashi (2009) showed that the bacteria in biofilm can accumulate high surfactin concentrations, much more than when surfactin is produced by bacteria in planktonic conditions (Shahedur and Takashi 2009; Zohora et al. 2009; Shaligram and Singhal 2010). This study provided evidence of biofilm formation when activated carbon and attapulgite powder were added to Landy medium, with the addition of activated carbon significantly promoting the synthesis of surfactin but inhibiting iturin synthesis (p < 0.05). However, synthesis of both iturin and surfactin was significantly enhanced by the addition of attapulgite powder, with significant changes in iturin composition (p < 0.05). However, it is not known how activated carbon and attapulgite powder affect biofilm formation and subsequently the synthesis of different lipopeptides. This needs to be explored in the future to get a better understanding of the associated mechanisms.

The antimicrobial lipopeptides are synthesized by a non-ribosomal peptide synthetase from multiple modules by a method called “multi-carrier thiotemplate mechanism” (Schneider and Marahiel 1998; Koumoutsi et al. 2004). The sequence of amino acids in the product is determined by the sequence of the peptide synthetase modules (Stein 2005), and each module contains multiple functional domains. This synthesis mechanism of multicomponent enzyme complex system is affected by experimental conditions, and lipopeptides which contain multiple components and multiple isomers can be produced by the lipopeptides produce strains (Shaligram and Singhal 2010).

The culture medium, shaking speed of the rotary shaker, fermentation method and addition of a solid matrix with large specific surface area had a significant impact on the type, amount, and proportion of the homologue constituents of the lipopeptides produced by *B. natto* NT-6 strain (p < 0.05). Thus accumulation of the specific components of lipopeptides can be achieved by controlling the media and fermentation conditions resulting in the formation of products with specific properties and functions. This study lays the foundation for synthesis of multifunctional lipopeptide products from fermentation.

## Data Availability

Data will not be shared, because the following study need to be kept confidential.

## References

[CR1] Akpa E, Jacques P, Wathelet B, Paquot M, Fucks R, Budzikiewicz H, Thonart P (2001). Influence of culture conditions on lipopeptide production by *Bacillus subtilis*. Appl Biochem Biotechnol.

[CR2] Alonso S, Martin PJ (2016). Impact of foaming on surfactin production by *Bacillus subtilis*: implications on the development of integrated in situ, foam fractionation removal systems. Biochem Eng J.

[CR3] Andrade CJD, Andrade LMD, Bution ML, Pastore GM (2016). Optimizing alternative substrate for simultaneous production of surfactin and 2,3-butanediol *by Bacillus subtilis* LB5a. Biocatal Agric Biotechnol.

[CR4] Banat IM, Franzetti A, Gandolfi I, Bestetti G, Martinotti MG, Fracchia L, Smyth TJ, Marchant R (2010). Microbial biosurfactants production, applications and future potential. Appl Microbiol Biotechnol.

[CR5] Bie XM, Lu FX, Lu ZX, Huang XQ, Shen J (2006). Isolation and identification of lipopeptides produced by *Bacillus subtilis* fmbJ. Chin J Biotechnol.

[CR6] Chen WC, Juang RS, Wei YH (2015). Applications of a lipopeptide biosurfactant, surfactin, produced by microorganisms. Biochem Eng J.

[CR7] Deng Q, Wang W, Sun L, Wang Y, Liao J, Xu D, Liu Y, Ye R, Gooreratne R (2017). A sensitive method for simultaneous quantitative determination of surfactin and iturin by LC-MS/MS. Anal Bioanal Chem.

[CR8] Desai JD, Banat IM (1997). Microbial production of surfactants and their commercial potential. Microhiol Mol Biol Rev.

[CR9] Fahim S, Dimitrov K, Gancel F, Vauchel P, Jacques P, Nikov I (2012). Impact of energy supply and oxygen transfer on selective lipopeptide production by *Bacillus subtilis* BBG21. Bioresour Technol.

[CR10] Koumoutsi A, Chen XH, Henne A, Liesegang H, Hitzeroth G, Franke P, Vater J, Borriss R (2004). Structural and functional characterization of gene clusters directing nonribosomal synthesis of bioactive cyclic lipopeptides in *Bacillus amyloliquefaciens* strain FZB42. J Bacteriol.

[CR11] Marchant R, Banat IM (2012). Microbial biosurfactants: challenges and opportunities for future exploitation. Trends Biotechnol.

[CR12] Mnif I, Chaabouniellouze S, Ghribi D (2012). Optimization of the nutritional parameters for enhanced production of *B subtilis* SPB1 biosurfactant in submerged culture using response surface methodology. Biotechnol Res Int.

[CR13] Nitschke M, Ferraz C, Pastore GM (2004). Selection of microorganisms for biosurfactant production using agroindustrial wastes. Braz J Microbiol.

[CR14] Ohno A, Aao T, Shoda M (1995). Effect of temperature on production of lipopeptide antibiotics, iturin A and surfactin by a dual producer, *Bacillus subtilis* RB14, in solid-state fermentation. J Ferment Bioeng.

[CR15] Sandrin C, Peypoux F, Michel G (1990). Co-production of surfactin and iturin A, lipopeptides with surfactant and antifungal properties by *Bacillus subtilis*. Appl Biochem Biotechnol.

[CR16] Schneider A, Marahiel MA (1998). Genetic evidence for a role of thioesterase domains integrated in or associated with peptide synthetases, in non-ribosomal peptide biosynthesis in *Bacillus subtilis*. Arch Microbiol.

[CR17] Shahedur RM, Takashi A (2009). Production characteristics of lipopeptide antibiotics in biofilm fermentation of *Bacillus subtilis*. J Environ Sci.

[CR18] Shaligram NS, Singhal RS (2010). Surfactin-A review on biosynthesis, fermentation, purification and applications. Food Technol Biotechnol.

[CR19] Stein T (2005). *Bacillus subtilis* antibiotics: structure, syntheses and specific functions. Mol Microbiol.

[CR20] Sun L, Wang Y, Liu H, Xu D, Zhang Y, Nie F (2013). Identification of antimicrobial lipopetides component produced by isolate from douchi and its antimicrobial properties. China Biotechnol.

[CR21] Thompson DN, Fox SL, Bala GA (2001). The effect of pretreatments on surfactin production from potato process effluent by *Bacillus subtilis*. Appl Biochem Biotechnol.

[CR22] Zohora US, Rahman MS, Ano T (2009). Biofilm formation and lipopeptide antibiotic iturin A production in different peptone media. J Environ Sci.

[CR23] Zohora US, Rahman MS, Khan AW, Okanami M, Ano T (2013). Improvement of production of lipopeptide antibiotic iturin A using fish protein. J Environ Sci.

